# A model combining nocturnal baseline impedance, post-reflux swallow-induced peristaltic wave, and acid exposure time enhances prediction of proton pump inhibitor response for extraesophageal symptoms in a Chinese population

**DOI:** 10.3389/fmed.2026.1720817

**Published:** 2026-03-16

**Authors:** Rong Wang, Gengzhao Guo, Jianzhen Li, Yongxiu Lin, Dazhou Li, Linfu Zheng, Gang Liu, Yulong Li, Wen Wang

**Affiliations:** 1 Department of Gastroenterology, The Affiliated People’s Hospital of Fujian University of Traditional Chinese Medicine, Fuzhou, China; 2 Department of Gastroenterology, 900th Hospital of People’s Liberation Army, Fuzhou, China; 3 Department of Gastroenterology, Fuzong Clinical Medical College of Fujian Medical University, Fuzhou, China; 4 Department of Gastroenterology, Jinjiang Municipal Hospital, Shanghai Sixth People's Hospital Fujian Campus, Quanzhou, China; 5 Health Management Center, The Second Affiliated Hospital of Fujian University of Traditional Chinese Medicine, Fuzhou, China

**Keywords:** acid exposure time, extra-oesophageal symptoms, gastroesophageal reflux disease, mean nocturnal baseline impedance, post-reflux swallow-induced peristaltic wave index

## Abstract

**Background:**

Gastroesophageal reflux disease(GERD) with extraoesophageal symptoms tends to respond poorly to proton pump inhibitors (PPIs).

**Aims:**

This study aimed to evaluate the role of mean nocturnal baseline impedance (MNBI) and post-reflux swallow-induced peristaltic wave (PSPW) index in predicting PPIs response among GERD patients with extraoesophageal symptoms.

**Methods:**

We investigated the predictive significance of clinical, endoscopic, and pre-treatment 24-h impedance-pH monitoring data in determining PPIs response, evaluating both the individual and combined predictive capacities of MNBI and PSPW index.

**Results:**

A total of 92 Chinese patients were analyzed, revealing pathological MNBI (*p* = 0.013, OR = 4.217, 95%CI: 1.359–13.089), PSPW index (*p* = 0.015, OR = 4.026, 95%CI: 1.308–12.396), AET (*p* = 0.017, OR = 4.246, 95%CI: 1.290–13.982), and typical GERD symptoms (*p* = 0.040, OR = 3.467, 95%CI: 1.057–11.374) as independent predictors of PPIs response. Notably, the combined AUC of MNBI, PSPW index, and AET was significantly higher than that of the individual parameters (*p* < 0.05). Among patients with normal AET, the combined AUC of MNBI and PSPW index was significantly higher than that of MNBI alone (*p* < 0.001). In patients with only extra-oesophageal symptoms, the AUC of the MNBI and the combined parameters of the three indices were higher than that of the AET (*p* < 0.05).

**Conclusion:**

MNBI and PSPW index serve as independent predictors of PPI response in GERD patients with extraesophageal symptoms, including those with normal AET. Importantly, their combined use significantly enhances predictive performance. MNBI remains a valuable predictor in patients with only extraesophageal symptoms. This study conducted a preliminary investigation into the predictive role of the combined MNBI-PSPW model in a Chinese population, highlighting the clinical importance of impedance parameter integration in managing extraesophageal GERD.

## Introduction

Gastroesophageal reflux disease (GERD) is characterized by the reflux of stomach content into or beyond the oesophagus, including the lungs, which can cause a range of symptoms and end-organ complications (1). The extraoesophageal symptoms of GERD can develop through direct mucosal contact with gastric refluxate, or via indirect neurogenic or immune-mediate mechanisms (2). Extraoesophageal GERD symptoms are often more difficult to treat compared to typical GERD (3, 4). The role of acid suppression therapy is often limited given that these symptoms are primarily associated with non-acid reflux (5–9). Accurate screening and identification of patients with extraoesophageal symptoms who may benefit from acid suppression therapy is thereby essential to improve patient outcomes.

Recent years have seen the development of novel oesophageal impedance-pH parameters, namely mean nocturnal baseline impedance (MNBI) and post-reflux swallow-induced peristaltic wave (PSPW) index for the diagnosis of GERD. The 2018 Lyon Consensus on GERD has recommended such indices as an adjunct in cases of inconclusive impedance-pH monitoring results (10). MNBI reflects oesophageal acid exposure time and mucosal integrity (11, 12), while PSPW index assesses the efficacy of oesophageal chemical clearance. MNBI has been useful in assisting the diagnosis and prediction of acid suppression therapy response among patients with typical non-erosive reflux disease (7, 13–15). The diagnostic and predictive role of PSPW index in pathological reflux patients has also been shown (16, 17). The upper oesophagus, pharynx, and airway mucosa are often more sensitive to weak or non-acid reflux compared to the oesophagus (18–21), resulting in the characteristic features of lower-to-normal AET, less total reflux events, and non-acid reflux as the predominant pathological presentation (6, 22). However, reports on the role of novel impedance-pH parameters in predicting acid suppression therapy response in extraoesophageal GERD remains limited.

This study thereby aimed to assess the value of pre-treatment novel oesophageal impedance-pH parameters, MNBI and PSPW index, in predicting response to proton pump inhibitors (PPIs) among patients with extraoesophageal symptoms. A preliminary investigation on the predictive ability of MNBI, PSPW and AET as individual and combined parameters was further conducted.

## Materials and methods

### Study design and subjects

A retrospective cohort study study involving patients with a proposed diagnosis of extraoesophageal GERD treated with standard PPI therapy at the Department of Digestive Diseases of 900th Hospital of People’s Liberation Army between June 2021 and October 2022 was conducted.

The inclusion criteria were as follows: (1) presence of extraoesophageal symptoms (such as globus sensation, cough, repetitive throat clearing, dry throat, sore throat, hoarseness, and excessive phlegm, among others) with or without typical GERD symptoms (defined as reflux or heartburn); (2) pathological reflux or extra-esophageal symptoms associated with reflux as suggested during 24-h esophageal impedance-pH monitoring;(3) presence of pre-treatment 24-h oesophageal impedance-pH monitoring results and completion of standard PPI therapy (10 mg rabeprazole twice daily for at least 12 weeks) following definitive diagnosis. The exclusion criteria were as follows: (1) age <18 years or >70 years; (2) failure to discontinue relevant medications prior to impedance-pH monitoring (including PPIs/H2-receptor antagonist <7 days, gastrointestinal stimulants/magnesium and aluminium acid preparations <3 days); (3) presence of severe comorbidities; (4) history of surgery or trauma to the larynx, neck, and gastrointestinal tract; (5) possible alternative cause for extraoesophageal symptoms (including post-nasal drip, asthma, respiratory infections, and adverse drug reactions); (6) suspected eosinophilic esophagitis on gastroscopy and pathological examination; and (7) suspected oesophageal dysmotility disorders on upper gastrointestinal imaging or high-resolution manometry.

Baseline clinical characteristics, endoscopic findings, and impedance-pH results were retrospectively collected in all patients. In accordance with prior literature (23, 24), no stringent inclusion criteria were applied regarding the duration of symptoms. Baseline and post-treatment severity of extraoesophageal symptoms, defined as the intensity of symptoms during an episode, were assessed using the visual analogue scale (VAS). Patients with symptom improvement ≥ 50% on the VAS were classified as PPI responders (2, 25).

### Endoscopic evaluation

Laryngoscopy was performed for the diagnosis of laryngopharyngeal reflux. The reflux finding scale (RFS) was used, and a positive diagnosis was considered as RFS score > 7 (26).

Esophagoduodenoscopy was performed for the evaluation of reflux esophagitis (RE) and the gastro-oesophageal flap valve (GEFV). RE was graded using the Los Angeles standard classification (LA-A–LA-D). GEFV was assessed using the Hill classification, with Grades I and II defined as normal, and Grades III and IV as abnormal.

### 24-h oesophageal impedance-pH monitoring

A portable oesophageal impedance-pH monitoring system (OMOM, Jinshan Technology Group Co, Ltd., Chongqing, China) and a monitoring catheter (model JSIpC-8Z1P-57) were used. Six impedance channels were positioned 3 cm (Z6), 5 cm (Z5), 7 cm (Z4), 9 cm (Z3), 15 cm (Z2), 17 cm (Z1) above the lower oesophageal sphincter (LES), and pH electrodes placed 5 cm (pH1) above the LES. After 24 h of monitoring, the data were uploaded to a dedicated software (JSIpDS-1, V2.0.8.1, Jinshan Technology Group Co., Ltd., Chongqing, China). All data were first automatically analysed by the software, and were then reviewed by two physicians.

Standard impedance-pH parameters were collected, including the total number of reflux, with >80 reflux events defined as pathological, and the number of proximal reflux. Acid exposure time (AET) was defined as the percentage of total recording time with exposure to pH < 4. AET > 4.2% was considered pathological, as per the recent China and Asia-Pacific National expert consensus suggesting a population difference in optimal diagnostic cut-offs for AET (27, 28). Symptom index (SI) was defined as percentage of the total number of symptoms associated with reflux, with SI ≥ 50% considered pathological. Symptom association probability (SAP) was defined as the correlation between symptoms and reflux, with SAP≥95% considered pathological (29).

In terms of novel impedance-pH parameters, MNBI was collected from the farthest impedance channel (Z6, originally reported for the distal-most channel at 3 cm above the LES), and calculated using data of 3 10-min time periods during the night-time recumbent period (about 01:00, 02:00, and 03:00 a.m.) (14). In this study, MNBI refers specifically to distal MNBI. MNBI≤2,291 *Ω* was considered pathological.10 Proximal MNBI was calculated as the average of MNBI values from the channels located at 15 and 17 cm above the LES (15). PSPW index was assessed by manual labelling of PSPWs followed by automatic calculation using a dedicated software. PSPW index≤61% was considered pathological (10).

### Statistical analysis

Normally distributed continuous variables are expressed as mean ± standard deviation (x ± s) and were compared using independent samples t-test; while non-normally distributed variables are expressed as median(first quartile, third quartile) [M(P25, P75)] and were compared using the Mann–Whitney U test. Categorical variables are expressed as number (percentage) [n (%)] and were compared using the chi-square test. The predictors of PPI response were determined using the dichotomous logistic regression analysis. The combined parameter was obtained using the formula constructed from the intercept values and β coefficients of each parameter in the logistic regression. Predictive performance was assessed in terms of the area under the receiving operating characteristic (ROC) curve (AUC) and 95% confidence interval (95%CI). AUCs were compared using the Delong test.

All statistical analyses were performed using SPSS 25.0 (Armonk, NY, USA), and MedCalc 20.0. (MedCalc Software bvba, Ostend, Belgium). Statistical significance was considered with *p* < 0.05.

## Results

### Overall study population characteristics

A total of 92 patients were included, of whom 42 were male (45.65%). The mean age was 46.29 ± 9.76 years, and the mean BMI was 22.83 ± 2.94 kg/m^2^. The mean RSI and RFS scores were 12.67 ± 4.39 and 9.16 ± 3.43, respectively. Typical reflux symptoms were observed in 63 cases (68.47%). On esophagoduodenoscopy, RE was observed in 23 patients (25.00%), of which 14 (15.21%) and 9 (9.79%) grade LA-A and LA-B, respectively. Abnormal GEFV was observed in 25 patients (27.16%).

The mean MNBI was 1829.65 Ω; the mean proximal MNBI was 1922.69 Ω. The mean PSPW index was 47.79%. The median AET was 3.30%. Pathological MNBI, PSPW index, and AET were observed in 50 (54.3%), 54 (58.7%), and 32 (34.8%) patients, respectively. Positive SI and/or SAP was seen in 65 (70.6%) patients. The median total number of reflux episodes was 55.50, while the median number of acid and non-acid reflux episodes were 23.00 and 26.50, respectively. The median number of proximal reflux episodes was 23.00.

### Clinical characteristics between the patient groups

The baseline clinical characteristics of the patient groups are summarized in Table 1. Significantly higher proportions of typical GERD symptoms were observed among the PPI responders (79.09% vs. 59.18%, *p* = 0.041). No statistical differences in terms of sex, age, BMI, or Reflux symptom index (RSI) score were observed between the groups.

**Table 1:** Comparison of clinical characteristics between the patient groups.

Characteristics	Responders (*n* = 43)	Non-responder (*n* = 49)	*P*
Male, *n*(%)	19(44.19)	23(46.94)	0.791
Age, $\bar{x}$ ± s	44.77 ± 9.80	47.63 ± 9.63	0.926
BMI, $\bar{x}$ ± s	22.28 ± 2.30	23.31 ± 2.86	0.092
RSI, $\bar{x}$ ± s	12.60 ± 4.13	12.73 ± 4.64	0.887
Typical symptoms, *n*(%)	34(79.07)	29(59.18)	0.041

### Endoscopic manifestations between the patient groups

The endoscopic findings of the patient groups are shown in Table 2. Significantly higher rate of RE was observed among the responders (34.88% vs. 16.33%, *p* = 0.040). No statistical differences in RFS score or GEFV grade were observed between the groups.

**Table 2:** Comparison of endoscopic features between the patient groups.

Characteristics	Responders (*n* = 43)	Non-responder (*n* = 49)	*P*
RFS, $\bar{x}$ ± s	8.91 ± 3.61	9.39 ± 3.28	0.506
RE, *n*(%)	15(34.88)	8(16.33)	0.040
Abnormal GEFV, *n*(%)	12(27.91)	13(26.53)	0.882

### Impedance-pH parameters between the patient groups

The MNBI and PSPW index of the patient groups are compared in Table 3. Significantly lower MNBI and PSPW index were demonstrated among the responders. Significantly higher rates of pathological MNBI, pathological PSPW index, and pathological AET were also demonstrated.

**Table 3:** Novel pH-impedance parameters between the patient groups.

Characteristics	Responders (*n* = 43)	Non-responder (*n* = 49)	*P*
Proximal MNBI, $\bar{x}$ ± s	1836.99 ± 660.51	1997.89 ± 588.98	0.220
MNBI, $\bar{x}$ ± s	1468.75 ± 501.98	2146.36 ± 553.76	<0.001
^a^MNBI+, *n*(%)	34(79.07)	16(32.65)	<0.001
PSPW, $\bar{x}$ ± s	34.52 ± 18.56	59.43 ± 12.98	<0.001
^b^PSPW+, *n*(%)	35(81.39)	19(38.78)	<0.001
MNBI+ and/or PSPW+, *n*(%)	40(93.02)	27(55.10)	<0.001

a Pathological distal MNBI.

b Pathological PSPW index.

The conventional impedance-pH parameters of the patient groups are shown in Table 4. Significantly higher AET and rate of pathologic AET were observed in the responders. Significantly higher number of acid refluxes, and significantly lower number of non-acid refluxes were observed as well.

**Table 4:** Comparison of conventional impedance-pH parameters between the patient groups.

Characteristics	Responders (*n* = 43)	Non-responder (*n* = 49)	*P*
AET, *M*(*P_25_*, P*_75_*)	5.50(3.30, 14.30)	1.60(0.50, 3.35)	<0.001
pathological AET, *n*(%)	25(58.14)	7(14.29)	<0.001
Reflux number, *M*(*P_25_*, P *_75_*)	51.00(36.00, 74.00)	59.00(40.00, 78.00)	0.385
Acid reflux, *M*(*P_25_*, P*_75_*)	32.00(14.00, 46.00)	21.00(8.00, 28.00)	0.005
Non-acid reflux, *M*(*P_25_*, P*_75_*)	20.00(9.00, 33.00)	41.00(17.00, 54.00)	<0.001
Proximal reflux, *M*(*P_25_*, P*_75_*)	22.00(17.00, 31.00)	23.00(17.00, 35.00)	0.664
SAP and/or SI+, *n*(%)	31(72.09)	34(69.38)	0.776

### Multifactorial analysis for the prediction of PPI response

Pathological MNBI (*p* = 0.013, OR = 4.217, 95%CI: 1.359–13.089), pathological PSPW index (*p* = 0.015, OR = 4.026, 95%CI: 1.308–12.396), pathological AET (*p* = 0.017, OR = 4.246, 95%CI. 1.290–13.982), and presence of typical GERD symptoms (*p* = 0.040, OR = 3.467, 95%CI: 1.057–11.374) were identified as independent predictors of PPI response in the overall cohort.

### Predictive performance of MNBI, PSPW index, and AET as individual and combined parameters

**Figure 1:**
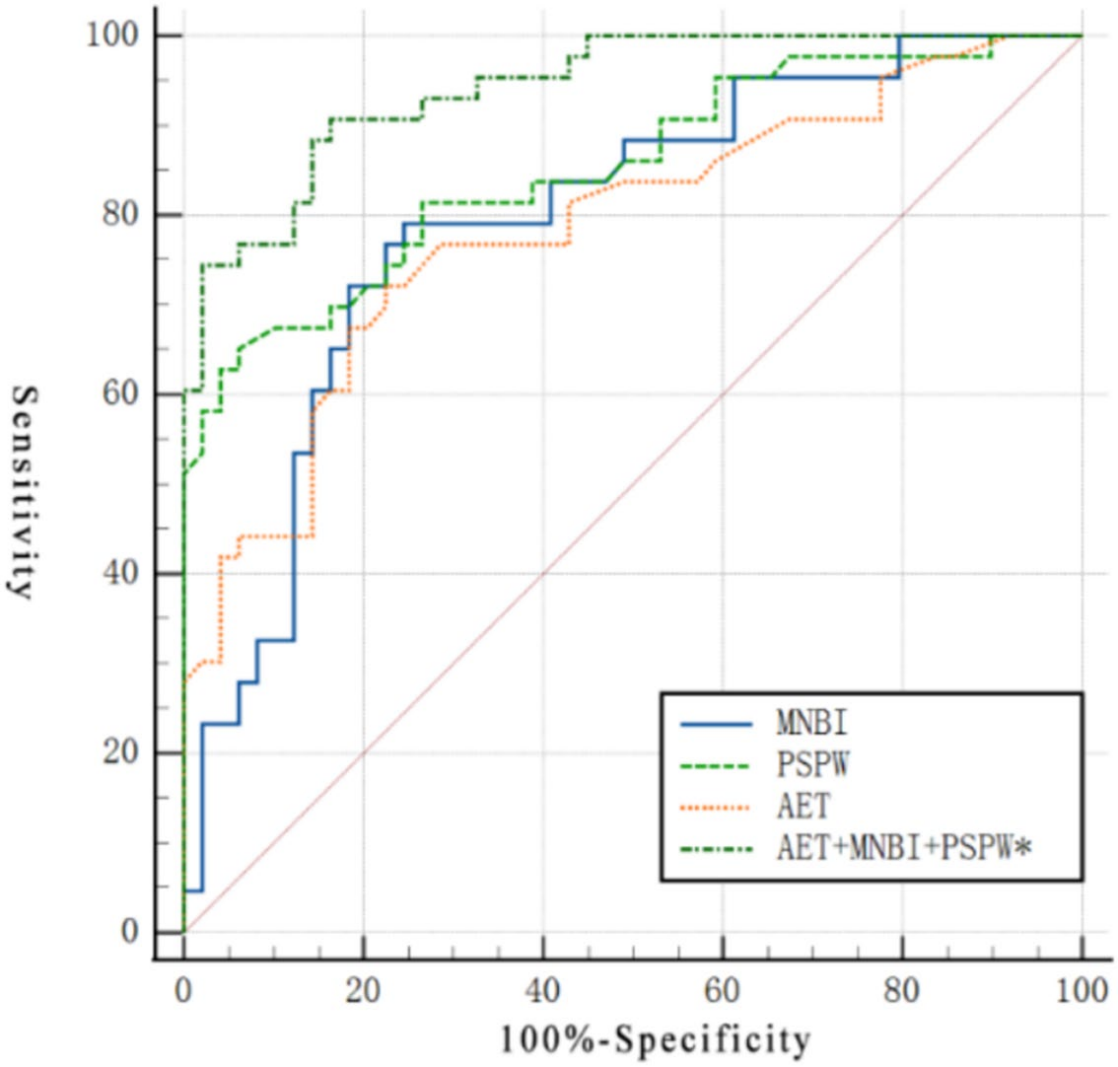
ROC curves of MNBI, PSPW index, and AET as individual and combined parameters for the prediction of PPI response. *The obtained formula of the model: y = 5.338–0.001*Log MNBI-0.074*LogPSPW+0.132 **×** LogAET, the probability (*p*) of responders is calculated as: *p* = 1/(1 + e^-y^).

The ROC curve analysis results of MNBI, PSPW index, and AET, both independently and combined, are shown in Figure 1. Individually, MNBI, PSPW index, and AET significantly associated with PPI response. The AUCs were 0.796 (95%CI: 0.699–0.873, *p* < 0.001), 0.853 (95%CI: 0.764–0.918, *p* < 0.001), and 0.786 (95%CI: 0.688–0.864, *p* < 0.001), respectively. No significant differences were observed between the AUCs of all 3 parameters (MNBI vs. AET, *p* = 0.868; PSPW vs. AET, *p* = 0.222; and MNBI vs. PSPW, *p* = 0.330). The AUC of the combined parameters was 0.942 (95%CI: 0.873–0.980, p < 0.001), which was significantly higher than that of each of the individual parameters (MNBI vs. combined parameters: *p* = 0.004; PSPW vs. combined parameters, *p* = 0.040; and AET vs. combined parameters, *p* = 0.003).

The optimal cutoff values of MNBI, PSPW, and AET for predicting PPI response in patients with extra-esophageal symptoms were ≤1874.83 *Ω*, ≤39.55%, and ≤3.45%, respectively. Various combinations of these cutoffs were applied to calculate the AUC, and only combinations yielding an AUC > 0.5 were considered. Among these, the combination defined by (1) MNBI ≤1874.83 Ω or AET ≤ 3.45%, and (2) PSPW ≤39.55% yielded the highest AUC. Details are provided in Table 5.

**Table 5:** AUC of parameter combinations using optimal cutoffs for predicting PPI response.

Variable	AUC	SE	95% CI
(1)	0.721	0.0479	0.645 to 0.832
(2)	0.703	0.0406	0.698 to 0.872
(1) and (2) and (3)	0.538	0.0287	0.431 to 0.643
(1) and (2)	0.689	0.0479	0.584 to 0.781
(1) or (2) and (3)	0.701	0.0471	0.624 to 0.814
(1) or (3) and (2)	0.807	0.0401	0.711 to 0.882

(1) MNBI≤1874.83 Ω; (2) PSPW≤39.55%; (3) AET≤3.45%.

### Sub-analysis of patients with normal AET

A sub-analysis involving patients with normal AET was performed. Pathological MNBI (*p* = 0.029, OR = 4.269, 95%CI: 1.156–15.764) and pathological PSPW index (*p* = 0.007, OR = 6.422, 95%CI: 1.645–25.075) remained as independent predictors of PPI response.

**Figure 2:**
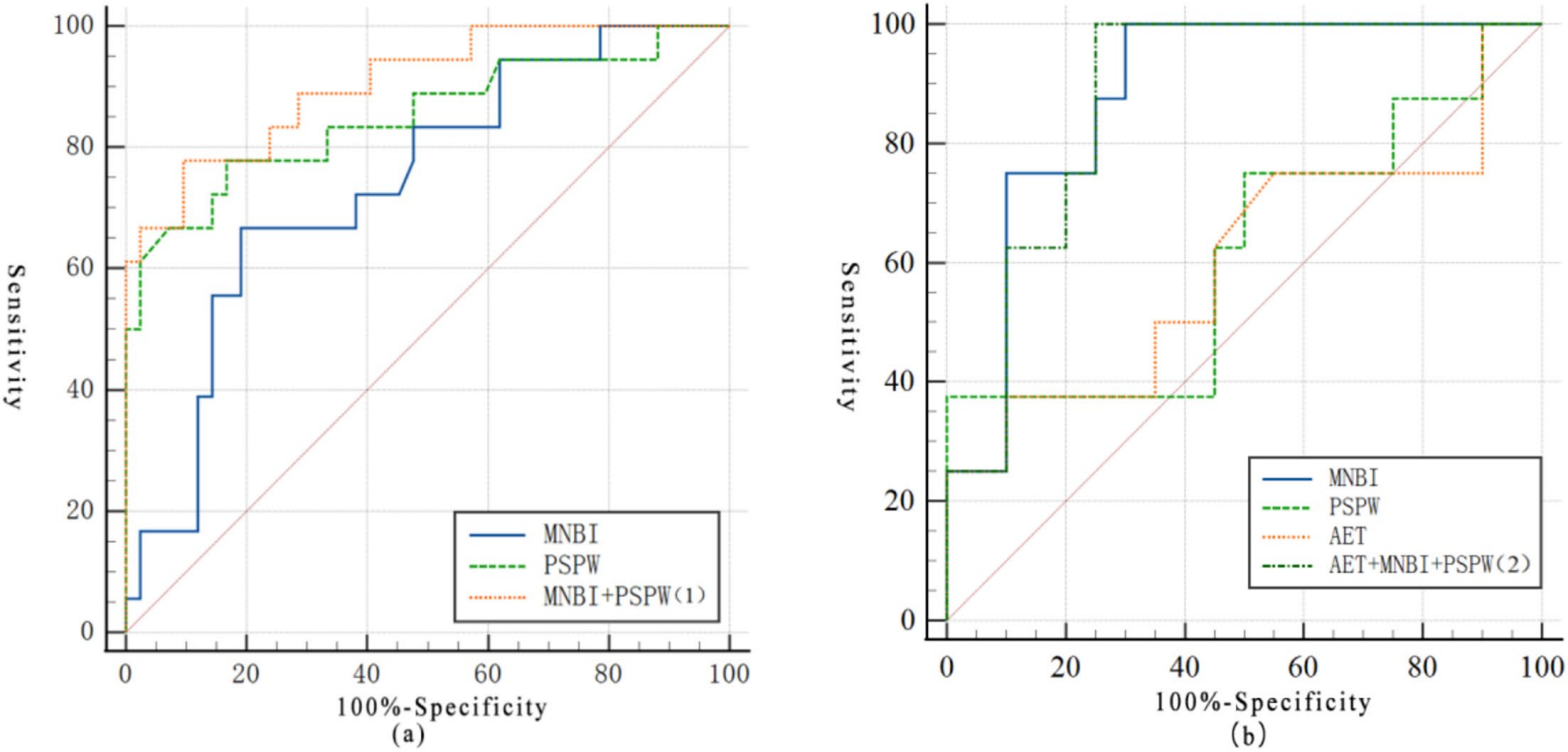
ROC curves of key models for predicting PPI response in subgroup **(a)** ROC curves of MNBI, PSPW index, and AET as individual and combined parameters for the prediction of PPI response **(b)** ROC curves of MNBI and PSPW index as individual and combined parameters for the prediction of PPI therapy in patients with normal AET. *The obtained formula of the model: (1) *y* = 8.395–0.02*Log MNBI-0.109*LogPSPW; (2) *y* = 4.872–0.003*Log MNBI-0.009*LogPSPW+0.009 **×** LogAET. The probability (*p*) of responders was calculated as: *p* = 1/(1 + *e^-y^*).

A sub-analysis on the predictive performance of the parameters in patients with normal AET (n = 60) was performed, as shown in Figure 2a. Both MNBI and PSPW index remained significantly correlated with PPI response. The AUCs were 0.740 (95%CI: 0.611–0.845, *p* < 0.001) and 0.850 (95%CI: 0.734–0.929, *p* < 0.001), respectively, with no significant difference observed (*p* = 0.266). The AUC of MNBI and PSPW index as a combined parameter was 0.905 (95%CI: 0.801–0.965, *p* < 0.001), which was significantly higher than that of MNBI (*p* = 0.024), but insignificantly different to that of PSPW (*p* = 0.137).

### Sub-analysis of patients with only extraesophageal symptoms

Among the 29 patients with only extra-oesophageal symptoms, MNBI values were significantly lower in responders than in non-responders, and the proportion of patients with pathological MNBI was significantly higher, as shown in Table 6. There were no statistically significant differences in clinical characteristics, endoscopic characteristics, PSPW index, MNBI and other conventional pH impedance parameters.

**Table 6:** Comparison of characteristics between responders and non-responders in patients with only extra-oesophageal symptom.

Characteristics	Responders (*n* = 9)	Non-responder (*n* = 20)	*P*
Male, *n*(%)	3(33.33)	8(40.00)	0.245
Age, $\bar{x}$ ± s	42.89 ± 10.73	46.10 ± 9.89	0.434
BMI, $\bar{x}$ ± s	20.94 ± 3.01	23.28 ± 3.30	0.082
RSI, $\bar{x}$ ± s	12.33 ± 3.50	11.60 ± 3.80	0.626
RFS, $\bar{x}$ ± s	11.33 ± 2.78	9.20 ± 3.24	0.099
RE, *n*(%)	3(33.33)	3(15.00)	0.339
Abnormal GEFV, *n*(%)	3(33.33)	6(30.00)	1
Proximal MNBI, $\bar{x}$ ± s	1719.69 ± 708.51	1852.14 ± 484.64	0.561
MNBI, $\bar{x}$ ± s	1409.15 ± 410.87	2046.52 ± 523.00	0.003
^a^MNBI+, *n*(%)	8(88.89)	8(40.00)	0.012
PSPW, $\bar{x}$ ± s	43.52 ± 21.53	55.14 ± 14.84	0.102
^b^PSPW+, *n*(%)	6(66.67)	10(50.00)	0.454
MNBI+ and/or PSPW+, *n*(%)	9(100.00)	13(65.00)	0.066
AET, *M*(*P_25_*, P *_75_*)	2.20(0.40, 11.70)	1.80(0.65, 7.03)	0.759
pathological AET, *n*(%)	3(33.33)	6(30.00)	1
Reflux number, *M*(*P_25_*, P *_75_*)	47.00(39.00, 58.50)	64.50(41.75, 78.00)	0.104
Acid reflux, *M*(*P_25_*, P *_75_*)	20.00(9.00, 33.00)	22.50(18.25, 45.00)	0.422
Non-acid reflux, *M*(*P_25_*, P *_75_*)	27.00(7.00, 37.00)	30.00(11.50, 55.75)	0.346
Proximal reflux, *M*(*P_25_*, P *_75_*)	20.00(15.00, 28.00)	23.00(12.50, 35.00)	0.333
SAP and/or SI+, *n*(%)	7(77.78)	14(70.00)	1

a Pathological MNBI.

b Pathological PSPW index.

Figure 2b shows the results of ROC curve analysis of MNBI, PSPW index and AET alone or combined in patients with only extra-oesophageal symptoms. MNBI remained significantly associated with PPI response (AUC = 0.833, 95% CI: 0.649–0.945, *p* < 0.001), while PSPW index (AUC = 0.656, 95% CI: 0.457–0.821, *p* = 0.216) and AET (AUC = 0.536, 95% CI: 0.343–0.722, *p* = 0.794) did not show significant correlation. The AUC of MNBI was significantly higher than that of AET (*p* = 0.034). The AUC of the combined parameters was 0.875 (95% CI: 0.695–0.969, p < 0.001), which was significantly higher than that of AET (*p* = 0.039). There was no statistically significant difference in the comparison of AUC for all of other parameters. (MNBI vs. PSPW, *p* = 0.064; PSPW vs. AET, *p* = 0.909; combined parameters vs. MNBI, *p* = 0.734; combined parameters vs. PSPW, *p* = 0.061).

## Discussion

In clinical practice, screening patients with extra-oesophageal symptoms from those who can benefit from acid suppression therapy remains a challenge. In this study, we found that the MNBI and PSPW index measured before treatment were predictive of the response to acid suppression therapy in patients with extra-oesophageal symptoms, and that the combined use of each parameter improved the predictive ability of each parameter.

The relationship between novel impedance-pH parameters and PPI therapy has been explored in the literature. The association of pathological AET, pathological MNBI, and pathological PSPW index in the distal oesophagus with PPI response has been reported in the studies by Ribolsi et al. involving patients with reflux-related chronic cough (31, 32). Li et al. (33) have also demonstrated the predictive value of MNBI and PSPW for reflux-related cough in a Chinese population. The association of MNBI and PSPW index with satisfactory PPI response has further been demonstrated among patients with a wider range of extraoesophageal symptoms (23). however, it should be noted that the impedance-pH parameters were collected following 6 months of PPI therapy. The study by Rinsma et al. demonstrated a considerable increase in distal baseline impedance following PPI therapy, reflecting recovery in mucosal integrity (34). However, studies assessing the predictive value of pre-treatment novel impedance-pH parameters for PPI response in patients with extraoesophageal symptoms remains limited. In addition, it remains unclear whether the combination of AET and novel parameters can improve the prediction of acid suppression treatment response in patients with extraoesophageal symptoms compared with each parameter alone.

In terms of clinical characteristics, our study found a significantly higher proportion of patients with concurrent typical symptoms among the PPI responders, and that the presentation of typical symptoms itself was an independent predictor of PPI response. Such findings are in corroboration with those of previous studies (4, 23). It may thus be reasonable to liken such concurrent typical GERD symptoms to acid-related GERD in terms of aetiology (35). The presence of RE on endoscopy was not an independent predictor for PPI response in our study. This is in contrast with the findings of Ribolsi et al., wherein Grades LA-C and LA-D RE were found to be independently predict PPI response among patients with reflux-related chronic cough. Such discrepancy may be related to the difference in patient demographic, as lower acid reflux burden has been shown among RE patients of the Chinese population. As reported in the study by Zhang et al. (36) majority of the patients with RE were of grades LA-A and LA-B, among whom only 35% were found with pathological acid exposure and met the diagnostic criteria for GERD. In our study, 25% of the patients had RE, and all of them were classified as LA-A or LA-B. This closely resembles the 7.5–27.6% incidence of RE reported in the studies by Kondo and Tokashiki et al., which explored the endoscopic findings of patients with extraoesophageal symptoms (37, 38). Further exploration of the association between RE and PPI response among patients of the Chinese population, specifically, is thereby warranted.

In terms of impedance-pH parameters, PPI responders were found to be characterized by significantly lower MNBI and PSPW index at baseline. Our study further found pathological MNBI, pathological PSPW index, and pathological AET as independent predictors of PPI response. These findings are consistent with those reported in patients with symptoms of heartburn and chronic cough (31, 39). In the sub-analysis involving patients with normal AET, pathological MNBI and pathological PSPW index remained independent predictors of PPI response, emphasizing the clinical value of such novel impedance-pH parameters in guiding the treatment of extraesophageal GERD. However, PSPW index is no longer recommended as an aid to GERD diagnosis in the Lyone 2.0, which proposes that PSPW index may be used mainly in studies of GERD phenotyping (40). The use of PSPW index is limited by the variability of normative thresholds and the calculation process is cumbersome (30, 41). The value of PSPW index in predicting patient response to PPI therapy has been validated in some studies (16, 23, 31). Therefore, further research on the application of the PSPW index in patients’ response to PPI treatment is worthwhile, especially in the study of patients with extra-oesophageal symptoms, where new thresholds need to be further established and the calculation optimized. In current clinical practice, the automated computation of PSPW by pH-impedance software has simplified its assessment. Furthermore, combining PSPW with other parameters may help reduce errors associated with its variability. While the association of low MNBI with delayed acid clearance and prolonged oesophageal acid exposure is well-established (42). It has been proposed that there is a notable correlation between the PSPW index and MNBI. The decreased chemical clearance (low PSPW index) is thought to result in oesophageal mucosal damage (low MNBI) due to prolonged contact with acidic or weakly acidic reflux (43). Others have, however, speculated that increased PSPW index occur with acid reflux due to vagal esophagosalivary reflex and activation of oesophageal mechanoreceptors (44). But the mechanism behind the low MNBI and low PSPW indices, which reflect a patient’s response to PPI therapy, is currently unclear.

According to Lyon 2.0, an MNBI <1,500 Ω supports GERD diagnosis, while >2,500 Ω argues against it (40). This 1,500 Ω cutoff is lower than Lyon 1.0’s 2,292 Ω and creates a diagnostic gray zone (1500–2,500 Ω) that risks underdiagnosis. However, the 2023 Chinese GERD guideline retains older cutoffs (45), and an Asian study found a higher MNBI threshold was needed (46). Therefore, this study adopts the Lyon 1.0 cutoff value. Interestingly, we did not find that proximal MNBI had a predictive value for the PPI response, which is inconsistent with previous relevant studies (24, 33). This discrepancy may be related to the reflex theory in the pathophysiology of extra-esophageal symptoms, whereby acidification of the distal esophagus can induce a vagal reflex leading to extra-esophageal manifestations (47). In such a scenario, the absence of acidic reflux reaching the proximal esophagus might not affect the MNBI in that region.

The correlation of MNBI and PSPW index with PPI response was further validated in our ROC analysis. Previous studies involving patients with symptoms of heartburn and reflux-related cough found significantly better predictive performance with MNBI and PSPW index compared to AET (16, 31). However, the AUCs of the 3 parameters were insignificantly different to each other in our study. This may, again, be related to the population-based differences in AET, as alluded to by a recent consensus analysis demonstrating lower AET among healthy Asian populations (2.7–3.2%) compared to those from Europe and the United States (5.3–7%) (41). Importantly, we found that MNBI, PSPW index, and AET achieved significantly improved predictive power as a combined parameter. Among patients with normal AET, the combined parameter with MNBI and PSPW index similarly achieved better predictive performance, with statistical significance demonstrated when compared to MNBI as an individual parameter. The lack of significant difference in prediction performance between the combined parameter and PSPW index may be related to the small sample size of the sub-analysis. We conducted a preliminary investigation into the role of combined parameters. However, constrained by the relatively small sample size of this study, the clinical significance of the combined parameter model for predicting PPI response in patients with extra-esophageal symptoms warrants further validation in larger-scale studies.

In this study, we calculated the optimal cutoff values for each parameter to predict PPI response in patients with extra-esophageal symptoms and explored their combined application. Our preliminary findings indicate that the combination defined by (1) MNBI ≤1874.83 *Ω* or AET ≤ 3.45%, and (2) PSPW ≤39.55% yielded the highest AUC. The combined use of these parameters may aid in identifying PPI responders. However, given the absence of a healthy control group and the limited sample size, these results should be interpreted as exploratory and require further validation.

Twenty-nine patients in this study had only extra-oesophageal symptoms. Due to the small sample size, we only performed one-way analyses and plotted ROC curves (AUC). We found that MNBI values were still at a low level in responders, and the proportion of patients with pathological MNBI was higher in this group. ROC curve analysis also demonstrated that MNBI was an effective method for identifying responders. A recent study found that distal oesophageal MNBI predicted pathological reflux in patients with only pharyngeal reflux symptoms (48). Patients presenting with only extra-oesophageal symptoms present a diagnostic and treatment challenge. The utilization of MNBI values may prove an effective means of overcoming this dilemma.

Our study had several limitations. First, this study was a single-center, retrospective study. Multicenter, prospective studies are thereby needed to verify our findings. Second, as the primary aim of this study was to assess the predictive value of pre-treatment novel impedance-pH parameters for PPI response, external testing of the combined predictive model was not performed, raising a significant risk of overfitting. Without validation, the reported AUCs likely overestimate real-world performance. Third, in line with previous studies, we did not impose strict restrictions on symptom duration; consequently, the disease duration in our cohort ranged from several weeks to several years, which may have introduced significant heterogeneity into the study population. Fourth, the size of the sub-analysis cohort was small.

In conclusion, MNBI and the PSPW index may serve as predictive parameters for PPI response among GERD patients with extraoesophageal symptoms, even in those with normal AET. Their use could potentially help inform the individualized management of these patients. Furthermore, combining MNBI, PSPW index, and AET might improve the prediction of PPI response, which could ultimately support more tailored patient management and contribute to enhanced quality of life.

## Data Availability

The raw data supporting the conclusions of this article will be made available by the authors, without undue reservation.
